# Functional MRI evaluation of hyperbaric oxygen therapy effect on hand motor recovery in a chronic post-stroke patient: a case report and physiological discussion

**DOI:** 10.3389/fneur.2023.1233841

**Published:** 2023-09-29

**Authors:** Merav Catalogna, Amir Hadanny, Yoav Parag, Moran Adler, Vicktoria Elkarif, Shai Efrati

**Affiliations:** ^1^Sagol Center for Hyperbaric Medicine and Research, Shamir (Assaf Harofeh) Medical Center, Zerifin, Israel; ^2^Sackler School of Medicine, Tel-Aviv University, Tel-Aviv, Israel; ^3^Sagol School of Neuroscience, Tel-Aviv University, Tel-Aviv, Israel

**Keywords:** stroke, fMRI, brain connectivity, rehabilitation, HBOT, upper extremity

## Abstract

**Introduction:**

Impairments in activities of daily living (ADL) are a major concern in post-stroke rehabilitation. Upper-limb motor impairments, specifically, have been correlated with low quality of life. In the current case report, we used both task-based and resting state functional MRI (fMRI) tools to investigate the neural response mechanisms and functional reorganization underlying hyperbaric oxygen therapy (HBOT)-induced motor rehabilitation in a chronic post-stroke patient suffering from severe upper-limb motor impairment.

**Methods:**

We studied motor task fMRI activation and resting-state functional connectivity (rsFC) in a 61-year-old right-handed male patient who suffered hemiparesis and physical weakness in the right upper limb, 2 years after his acute insult, pre- and post-treatment of 60 daily HBOT sessions. Motor functions were assessed at baseline and at the end of the treatment using the Fugl–Meyer assessment (FMA) and the handgrip maximum voluntary contraction (MVC).

**Results:**

Following HBOT, the FMA score improved from 17 (severe impairment) to 31 (moderate impairment). Following the intervention during trials involving the affected hand, there was an observed increase in fMRI activation in both the supplementary motor cortex (SMA) and the premotor cortex (PMA) bilaterally. The lateralization index (LI) decreased from 1 to 0.63, demonstrating the recruitment of the contralesional hemisphere. The region of interest, ROI-to-ROI, analysis revealed increased post-intervention inter-hemispheric connectivity (*P* = 0.002) and a between-network connectivity increase (*z*-score: 0.35 ± 0.21 to 0.41 ± 0.21, *P* < 0.0001). Seed-to-voxel-based rsFC analysis using the right SMA as seed showed increased connectivity to the left posterior parietal cortex, the left primary somatosensory cortex, and the premotor cortex.

**Conclusion:**

This study provides additional insights into HBOT-induced brain plasticity and functional improvement in chronic post-stroke patients.

## Introduction

Stroke is the second leading cause of long-term disability worldwide, where ~80% of individuals with acute stroke present upper-limb motor impairment, and 50–60% of them will have persistent disability or weakness at the chronic phase (1, 2). Impaired upper-limb motor function is highly associated with low self-care ability, limited mobility, and poor quality of life (3). Furthermore, subjective wellbeing was found to decrease 1 year after stroke and was mainly correlated with arm motor impairments (4). These long-term disabilities may affect social reintegration and result in direct and indirect economic impacts. Therefore, improving upper-limb function is a core element of stroke rehabilitation.

Biological mechanisms of motor function recovery were found to be associated with cell genesis, structural and functional neuroplasticity, and reorganization of neural pathways that were mostly observed within the first few months post-stroke (5–8). Therefore, formal rehabilitation protocols are focused on the acute-sub-acute phase (up to 6 months), and long-term disability treatment is less frequent in the late chronic phase (9). Current interventions to enhance upper-limb recovery in those early stages include physical, constraint-induced movement therapy and occupational therapy. However, their beneficial effect is limited in the chronic phase (8, 10).

In recent years, evidence has been accumulating about the neuroplasticity effects of hyperbaric oxygen therapy (HBOT). Importantly, HBOT was found to induce neuroplasticity in chronic stages, even years after brain injury (11–15). These findings are supported by preclinical and clinical studies, demonstrating HBOT's effects through multiple mechanisms including anti-inflammatory, mitochondrial function restoration, increased perfusion via angiogenesis and induction, proliferation, and migration of stem cells (16–20). In post-stroke chronic patients, HBOT-induced significant changes in neurological function, neurocognitive recovery (21–23), post-stroke depression (24), and sleep and quality of life (14, 23, 25). However, the specific effect of HBOT on upper-limb function has not been evaluated.

In the current case report, we used both task-based and resting state functional MRI (fMRI) tools to investigate the neural response mechanisms, and functional reorganization underlying HBOT-induced motor rehabilitation in a chronic post-stroke patient suffering from severe upper-limb motor impairment. The results were compared to a matched healthy control subject.

## Case description

### Clinical presentation

**Figure 1:**
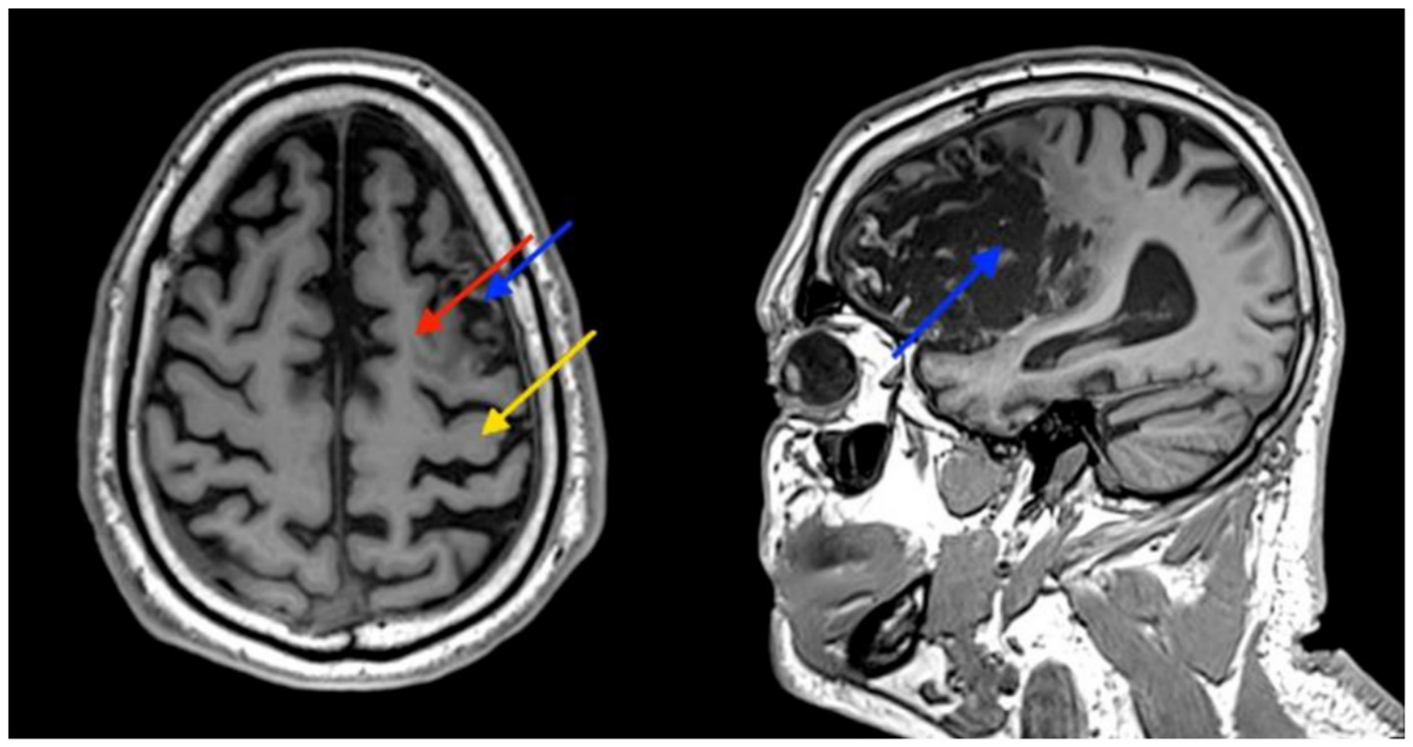
Patient's brain MRI. Images show atrophy and some damage to the supplementary motor cortex (SMA) (red arrow), and severe damage to the premotor cortex (PMA) (blue arrow). The primary motor cortex is relatively intact (yellow arrow).

A 61-year-old right-handed male patient presented to our center with right hemiparesis, speech, and cognitive impairments due to a left frontal chronic ischemic stroke, 2 years prior to his referral. The patient was conscious and comfortable and had co-existing adult-onset diabetes mellitus, hyperlipidemia, hypertension, and ischemic heart disease. During the sub-acute stroke period, he was treated daily for 4 months with physical, speech, and occupational therapy, followed by twice weekly treatment for a few more months. Additional clinical and demographic details are provided in Supplementary Table 1; Supplementary Figure 3. Despite therapy, he remained severely impaired and required assistance with activities of daily living (ADL), especially with fine motor function and communication and used a cane for walking. Anatomical MRI imaging showed a chronic infarct involving the supplementary motor cortex (SMA) and severe damage to the premotor cortex (PMA). The primary motor cortex was relatively intact (Figure 1).

An age-matched healthy (64-year-old, right-handed man) subject without a history of neurologic impairments or limitations in upper-limb movements participated for reference. The participants signed a written informed consent before inclusion. The fMRI study protocol was approved by Shamir Medical Center's Institutional Review Board (IRB) (No. 134-19-ASF).

### Therapeutic intervention

The HBOT protocol was administrated in a multi-place Starmed-2700 chamber (HAUX, Germany). The protocol comprised of 60 daily sessions, five sessions per week within a 2-month period. The HBOT protocol included breathing 100% oxygen by mask at 2ATA for 90 min with 5-min air breaks every 20 min. Compression/decompression rates were 1.0 m/min. During the therapeutic phase, the patient received physical and occupational therapy twice a week. The physical therapy regimen focused on lower-limb mobility, executing transitions between body positions, enhancing walking pace both with and without a cane, and proprioceptive training to augment coordination, reflexes, and balance. The occupational therapy incorporated activities of daily living (ADL) and instrumental ADL practice carried out both sitting and standing.

### Assessment tools

The motor function of the patient's affected upper limb was assessed at baseline and at the end of the HBOT session using the Fugl–Meyer assessment (FMA) and the handgrip maximum voluntary contraction (MVC).

The FMA contains 50 items to investigate upper (UE) and lower (LE) limb motor functions (26). The FM-UE test consists of movement instructions for the position (e.g., supination/pronation, flexion/extension, and adduction/opposition) of proximal, medial, and distal parts of the UL (i.e., shoulder, elbow, forearm, wrist, hand, and finger) as well as of tests for the existence and possibility to activate reflexes. The maximum FM-UE score is 66 points. An FM-UE score of 60–66 is defined as mild impairment. A score of 22–59 is considered moderate, and a score of 0–21 is considered severe impairment. The MVC test was measured as the mean force value when squeezing a handheld dynamometer for 2 s as strongly as possible.

### Brain imaging data acquisition

Brain imaging MRI scans were performed on a MAGNETOM VIDA 3T scanner, configured with 64-channel receiver head coils (Siemens Healthcare, Erlangen, Germany). The MRI protocol included 3D T2-weighted, 3D fluid-attenuated inversion recovery (FLAIR), susceptibility-weighted imaging (SWI), and high-resolution T1-weighted (MPRAGE).

Functional imaging data (task-fMRI) were acquired using gradient-echo (EPI) BOLD (blood oxygen level dependent) contrast sequence with a total of 198 volumes. Scan parameters were as follows: TR: 2,000 ms, TE: 30 ms, flip angle: 90°, voxel size: 2.0 × 2.0 × 3.0 mm, no gap, FOV: 205 mm^2^, number of slices: 45, and SMS factor: 3, axial slices parallel to the AP-PC plane.

A total of 300 volumes (7:40 min) of resting state fMRI scans were acquired using gradient-echo-planar imaging BOLD sequence. Scan parameters were as follows: TR: 1,500 ms, TE: 30 ms, flip angle: 90°, voxel size: 2.2 × 2.2 × 3.0 mm, distance factor: 25%, FOV: 210, number of slices: 36, and axial slices parallel to the AP-PC plane. During scanning, each participant was asked to remain still and relaxed with eyes open, without thinking of anything deliberate. Foam pads and earplugs were used to reduce head motion and scanning noise.

Structural T1-weighted MRI scans were acquired for co-registration purposes using a T1-weighted 3D magnetization-prepared rapid gradient-echo (MPRAGE) sequence in a sagittal plane with 1 mm isotropic resolution. Sequence parameters were as follows: TR: 2,000 ms, TE: 1.9 ms, flip angle: 9°, TI: 920 ms, FOV: 256 × 256, and 256 contiguous slices.

### Motor task fMRI

An fMRI motor task was performed both with the affected hand (AH) and the unaffected hand (UH) in a block design paradigm. During the task, participants were asked to press the index-finger button (ResponseGrip, NordicNeuroLab Inc., Norway) in response to a periodic visual cue—a flashing green dot either on the left or on the right side of the screen (frequency 0.5 Hz) (Supplementary Figure 1).

This block design paradigm consisted of ten alternating LEFT, RIGHT, and REST (fixation) blocks each lasting 30 s as illustrated in Supplementary Figure 2. NordicAktiva (NordicNeuroLab Inc., Norway, www.nordicneurolab.no), was used for stimuli presentation and performance accuracy acquisition. The task began with a 6 s countdown. Each active block consisted of 13 (600 ms ON, 1,400 ms OFF) trials. The active blocks began with an instruction statement presented for 4 s. The total experiment time was 6:36 min. Motor accuracy scores were calculated as the percentage of responses divided by the number of expected responses (13 × 4). Prior to the fMRI experiment, the task was explained, and participants practiced the motor task outside the scanner to familiarize themselves with the task and the grips.

### Motor task fMRI data analysis

Analysis of the time series BOLD data was performed using statistical parametric mapping software SPM12 (http://www.fil.ion.ucl.ac.uk/spm/) through a standard pre-processing procedure (27). All images were initially slice-time corrected, realigned, and resliced using a 6-parameter rigid-body spatial transformation to correct head motion and normalized to the MNI (Montreal Neurological Institute) space by using the unified segmentation normalization algorithm. Finally, spatial smoothing was performed using an 8 mm full-width half-maximum (FWHM) Gaussian kernel.

The general linear model was applied on the subject level. The design matrix incorporated the task and the six spatial axes of movement repressors. The task repressors were modeled as a boxcar function and were convolved with a canonical hemodynamic response function. A high-pass filter (cutoff of 128 s) was applied to account for slow signal drifts. Contrast images were thresholded at a significance level of a *P*-value of < 0.05, and familywise error (FWE) was corrected for multiple comparisons.

Motor function regions of interest (ROIs) were defined for each hemisphere based on activation likelihood estimation meta-analysis (28): primary motor cortex (M1), supplementary motor cortex (SMA), premotor cortex (PMA), primary somatosensory cortex (S1), posterior parietal cortex (PPC), and the cerebellum (CB). Masks were extracted using the WFU-PickAtlas Matlab toolbox (https://www.nitrc.org/projects/wfu_pickatlas/).

The level of activation and lateralization index (LI) was calculated for each ROI during left- and right-hand movements to identify changes in the cortical activation symmetry. LI was defined as (C – I)/(C + I), where C and I are the numbers of activated voxels in the contralateral and ipsilateral regions of the finger movement, respectively (29).

### Rs functional connectivity data analysis

RsFC analysis was carried out using the CONN-fMRI toolbox v17 as implemented using statistical parametric mapping software SPM12 (http://www.fil.ion.ucl.ac.uk/spm). Functional volumes of the pre-processing pipeline included slice-timing correction, realignment, co-registration, normalization-to-MNI space (152-brain template) with a resolution voxel size of 2 × 2 × 2 mm, and spatial smoothing (8 mm FWHM Gaussian kernel) steps (30). The pre-processing steps derived (1) the realignment covariate, containing the six rigid-body parameters characterizing the estimated subject motion, (2) the scrubbing covariate containing potential outlier scans performed with the CONNs artifact detection tool (ART), and (3) the quality assurance (QA) covariate based on global signal change (>3 standard deviations from the mean image intensity) and framewise displacement (FD) scan-to-scan head-motion. Age and sex were also used as group (second level) covariates. A component-based noise correction procedure (CompCor) approach (31) was used to identify additional confounding temporal factors controlling for physiological noise, BOLD signal present in WM, and head motion effects. Finally, residual BOLD time series were then bandpass filtered at a frequency range of 0.01–0.09 Hz (30). Individual connectivity maps were generated using the seed-to-voxel approach. We examined rsFC using a priori seeds derived from the FSL Harvard–Oxford atlas (32), focusing on large-scale brain networks related to the patient's reported symptoms, which included default mode (DMN), salience (SN), visual (VN), dorsal attention (DAN), fronto-parietal (FPN), language (LN), sensorimotor (SMN), and cerebellar (CN) (networks and the coordinates of the associated seed regions are presented in Supplementary Table 2). Bivariate correlation analysis was used to determine the linear association of the BOLD time series between the seed and significant voxel clusters. Fisher's *Z* transformation was applied to the correlation coefficients to satisfy normality assumptions. Then, rsFC maps were thresholded at a *P*-value of < 0.05, with familywise error (FWE) corrected for multiple comparisons. Adjusted ROI-to-ROI network analysis (33) was used to derive patient-specific adjusted ROIs as follows: in each map, a cluster was identified within spheres of 6 mm radii centered on the coordinates of interest from each network. Then, the voxel with the maximal value within the sphere was identified as the adjusted ROI. For each corrected location, the mean *Z*-score value was calculated within a 3 mm radius, producing a symmetrical 32 × 32 connectivity matrix. Whole brain within and between-network connectivity as well as inter- and intra-hemispheric values were calculated. Analysis was performed using in-house software written in MATLAB R2021b (MathWorks, Natick, MA).

### Statistical analysis

In addition to the MRI statistical methods, due to the single-subject nature of the study, brain global parameters were chosen to demonstrate the longitudinal change. Two-tailed paired *t*-tests were performed to compare connectivity variables between sessions when a normality assumption was held according to a Kolmogorov–Smirnov test. Data analysis was performed using MATLAB R2021b (MathWorks, Natick, MA) statistics and machine learning toolbox.

## Results

### Functional evaluation

Supplementary Table 3 shows the scores of the upper-limb motor function tests at pre- and post-intervention time points. The baseline FM-UE score was 17, indicating severe impairment. The post-intervention score was 31, considered as moderate impairment, and the 1-year follow-up score was 41, which is higher than the post-treatment score. At baseline, the patient was unable to perform the MVC test, while in post-intervention, he completed the maneuver with a score of 3 kg. The motor accuracy score, measured during the motor task fMRI, before treatment was 27% and improved to 100% after HBOT. Finally, the patient reported an improvement in life satisfaction and participation in the community as assessed by the short form-stroke impact (SF-SIS) scale (34) from 17/40 at baseline to 29/40 after 12 months of followup.

### Motor task-fMRI evaluation

**Figure 2:**
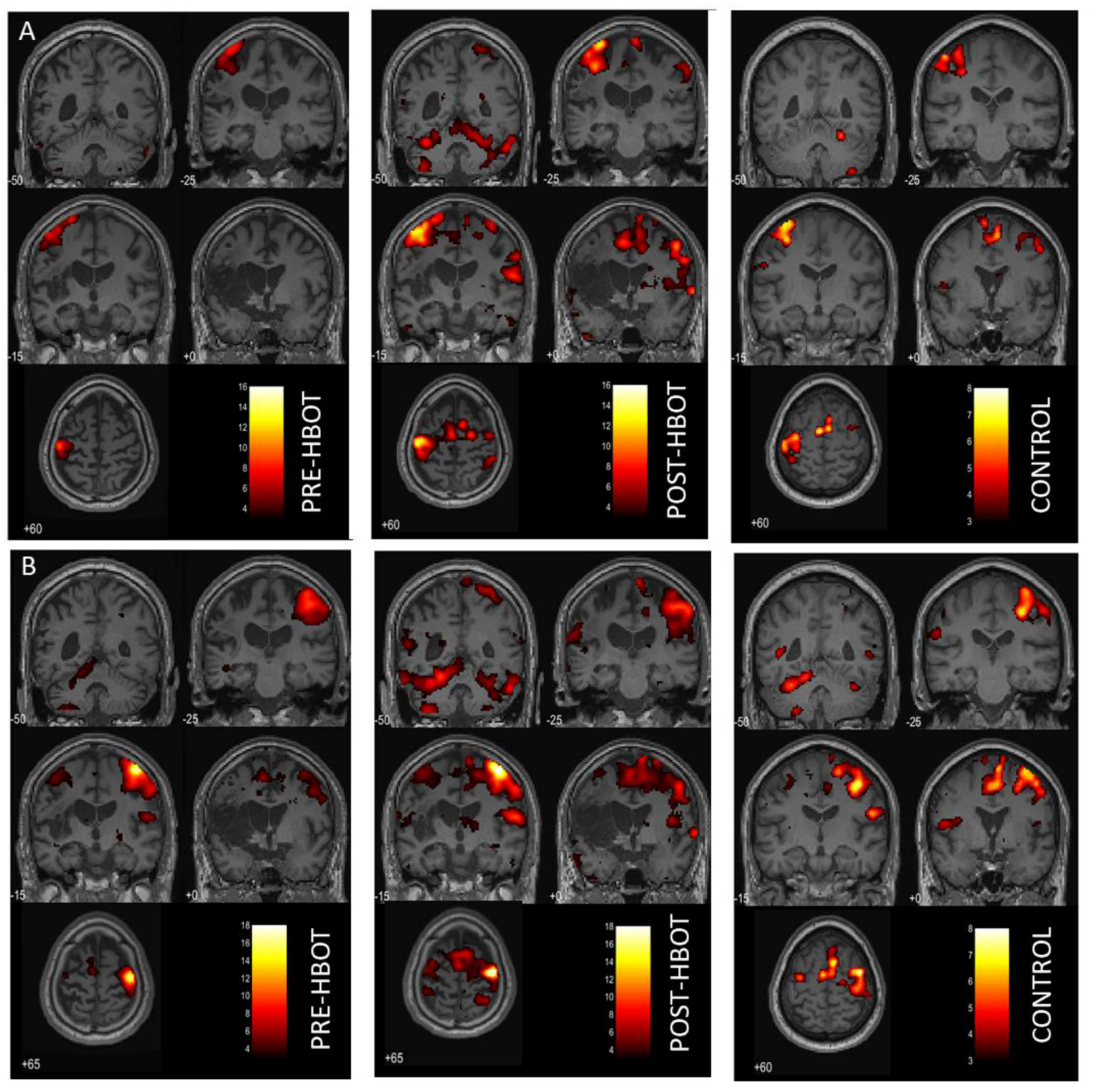
Motor task fMRI activation maps, pre- and post-HBOT. **(A)** Right finger movement (affected hand) > REST. **(B)** Left finger movement (unaffected hand) > REST. Compared to a healthy control subject. *P*_FWE_ < 0.05.

The whole-brain activation maps (affected and unaffected hand activation > rest conditions) at baseline and post-intervention session are shown in Figure 2 and Supplementary Table 4 (*P* < 0.05, FWE corrected). The overall post-intervention activation was increased during both the affected and unaffected finger movement. The quantification of ROI analysis is presented in Table 1.

**Table 1:** Motor task fMRI—ROI activation maxima.

**ROI**	**Stroke pre-HBOT**						**Stroke post-HBOT**						**HC**					
	***K***	*P*_FEW_	***T***	***X***	***Y***	***Z***	***K***	*P*_FEW_	***T***	***X***	***Y***	***Z***	***K***	*P*_FEW_	***T***	***X***	***Y***	***Z***
**L(UH)** > **REST**																		
SMA L	99	0.000	6.78	−6	−2	56	307	0.000	7.87	−2	−6	60	107	0.000	6.47	−4	−6	60
PMA L	113	0.000	7.48	−42	−10	52	119	0.000	6.75	−42	−10	52	–					
CB L	–						2,115	0.000	13.46	−14	−64	−18	258	0.000	7.00	−16	−54	−18
M1 R	268	0.000	14.53	42	−16	60	295	0.000	17.25	42	−14	60	125	0.000	7.32	40	−16	52
S1 R	490	0.000	11.50	44	−20	60	471	0.000	11.92	58	−20	46	–					
SMA R	–						636	0.000	8.23	10	2	76	357	0.000	6.89	8	10	58
PMA R	967	0.000	17.18	42	−16	64	1,244	0.000	18.83	40	−16	64	499	0.000	7.74	38	−14	50
CB R	–						770	0.000	9.51	26	−74	−18	–					
**R(AH)** > **REST**																		
M1 L	81	0.000	8.41	−38	−22	66	204	0.000	13.43	−44	−16	60	55	0.000	6.59	−34	−18	54
S1 L	110	0.000	9.31	−46	−20	60	246	0.000	13.58	−46	−20	60	–					
SMA L	–						328	0.000	10.52	−4	−6	54	103	0.000	7.53	−6	−6	60
PMA L	442	0.000	9.86	−30	−20	74	1,143	0.000	15.66	−32	−20	70	308	0.000	7.11	−34	−12	68
CB L	–						633	0.000	13.69	−24	−64	−18	–					
SMA R	–						162	0.000	8.31	8	8	46	82	0.000	6.67	6	−2	60
PMA R	–						282	0.000	10.51	48	0	48						
CB R	–						1,579	0.000	10.85	24	−76	−18	24	0.010	6.08	22	−54	−20

M1, primary motor cortex, SMA, supplementary motor area, PMA, premotor cortex, S1, primary somatosensory cortex, CB, cerebellum.

*P*_FWE_ < 0.05, *K* > 50; L, left, R, right, FWE, familywise error, UH, unaffected hand, AH, affected hand, HC, healthy control, *X, Y, Z* MNI coordinates.

During the affected hand trials (Figure 2A), post-intervention, the most substantial increases were found in the ipsilesional SMA and the PMA from *k* = 0 to 328, *T* = 10.52, and from *k* = 442 to 1,143, *T* = 15.66, respectively. At baseline, contralesional activation was not observed. However, post-intervention activation was observed in the contralesional SMA, *k* = 162, *T* = 8.31, and in the contralesional PMA, *k* = 328, *T* = 10.51. Activation in the left and right cerebellum was also observed (*k* = 633, *T* = 13.69, and *k* = 1,579, *T* = 10.85, respectively). LI was decreased (from 1 to 0.63, compared to 0.71 in HC), demonstrating recruitment of the contralesional hemisphere.

During the unaffected finger trials (Figure 2B), post-intervention, the most substantial increases were found in the ipsilesional SMA, from *k* = 99 to 307, *T* = 7.87, and contralesional SMA from *k* = 0 to 636, *T* = 8.23. However, LI was not markedly changed (from 0.78 to 0.72 compared to 0.77 in HC).

### RsFC evaluation

**Figure 3:**
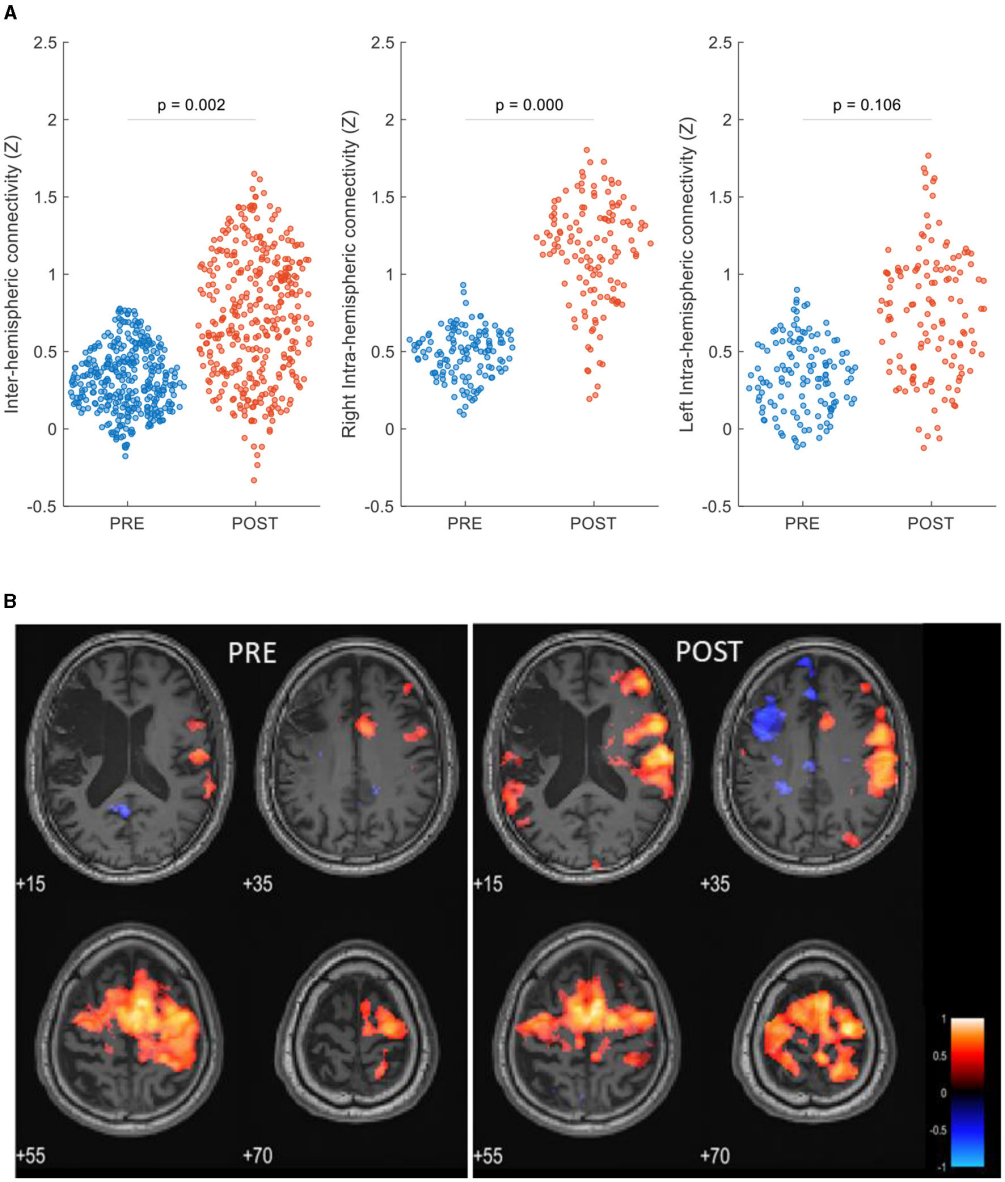
rsFC maps pre- and post-HBOT of a post-stroke patient. **(A)** ROI-to-ROI network analysis, swarm scatterplot of longitudinal changes in rsFC *z*-score connectivity in brain global network parameters. **(B)** Seed-to-voxel connectivity maps of longitudinal group differences, seed: right supplementary motor cortex (6, −3, 58). *P* < 0.05, FEW corrected, R, right.

The ROI-to-ROI analysis revealed increased post-intervention inter-hemispheric connectivity (*P* = 0.002). Post-treatment intra-hemispheric connectivity was increased in the contralesional hemisphere (*P* < 0.0001) and was not significantly changed in the ipsilesional hemisphere (*P* = 0.105) (Figure 3A). Network analysis showed that the patient's within-network connectivity was not significantly changed (*z*-score: 0.49 ± 0.24 to 0.50 ± 0.23, *P* = 0.47), while between-network connectivity was increased following treatment (*z*-score: 0.35 ± 0.21 to 0.41 ± 0.21, *P* < 0.0001).

Figure 3B shows seed-to-voxel-based rsFC maps and refers to the right SMA seed (6, −3, 58). Within the motor cortex, post-intervention increased connectivity was demonstrated in the left posterior parietal cortex (BA7, *z*-score: 0.23–0.61), the left primary somatosensory cortex (BA1, *z*-score: 0.36–0.64), the left premotor cortex (BA6, *z*-score: 0.56–0.78), and the right primary motor cortex (BA4, *z*-score: 0.50–0.69).

## Discussion

In this case study, we identified longitudinal changes in cerebral rsFC and activity related to motor recovery in a chronic post-stroke patient. We found a possible HBOT-induced reorganization in inter-hemispheric connectivity patterns and recruitment of bilateral SMA and PMA activation to improve motion control in the affected hand.

Functional motor-related neuroimaging was used in several studies to investigate the mechanisms of motor recovery after stroke (35–37). Since the functional cortical representation of the hand area is highly reproducible in healthy subjects, changes may reflect reorganization and cerebral plasticity (38). Cramer showed that activation volume in the primary sensorimotor-premotor cortex was 37% of the volume activated in patients with full recovery (39). In this study, improved motor skills were associated with increased volume activation, primarily in bilateral SMA and PMA and the cerebellum during affected hand activation, along with a decrease in LI, demonstrating recruitment of the contralesional hemisphere. Activation of both ipsilesional and contralesional somatosensory and PMC areas was previously found to be involved in cortical plasticity and successful motor function recovery (40, 41). In addition, contralesional cerebellar activity was related to the functional reorganization of the motor network after recovery (28).

Notably, several studies showed that good motor recovery after rehabilitation is correlated to near-normal activation patterns, where an increased volume activation and decreased LI are more likely to occur during the early post-stroke phase (36, 42, 43). Nevertheless, the activation volume depends on stroke severity and location, time from injury, type of rehabilitation, and differences in task or stimulus (44).

In the current study, motor dysfunction was attributed to severe damage in the secondary cortical areas, responsible for motor coordination and control, rather than the primary motor cortex. Therefore, remapping and recruitment of the contralateral cortex may replace the injured ipsilateral premotor neurons.

To strengthen the observation of changes in brain plasticity, we identified changes in post-intervention function connectivity. As rsFC was found to be dynamic over the recovery period (45), inter-hemispheric connectivity was found to be significantly reduced in the acute stage and become more balanced with recovery (46–49). Our results show that increased inter-hemispheric connectivity as well as between-network connectivity, demonstrating a change in the recruitment pattern of brain regions, may explain the patient's motor skill relearning. These results, acquired in separate scanning days, also support the increased activation in the contralesional hemisphere shown during motor task fMRI.

The observed clinical improvement at the delayed chronic phase in the presented patient, corroborated by findings in the fMRI, further supports the mechanism of action of the newly adopted protocol of HBOT. It has become evident that the synergistic effect of both hyperoxia and hyperbaric pressure leads to a significant enhancement in tissue oxygenation. This targets both oxygen-sensitive and pressure-sensitive genes, resulting in optimized mitochondrial metabolism with anti-apoptotic and anti-inflammatory effects (16, 17, 50). The intermittent increase in oxygen concentration triggers many of the mediators and cellular mechanisms usually induced during hypoxia but without the harmful effects of hypoxia itself. This phenomenon is known as the hyperoxic-hypoxic paradox (HHP) (16, 50).

Among other biological effects, intermittent hyperoxic exposure during HBOT can influence HIF-1 levels, matrix metalloproteinases (MMP) activity, and VEGF. It can induce stem cell proliferation, increase circulating levels of endothelial progenitor cells (EPCs), and factors related to angiogenesis, as well as promote angiogenesis itself and enhance blood flow in ischemic areas (16, 17, 50). Based on previous clinical studies involving brain single photon emission computerized tomography (SPECT) imaging, the beneficial impact of HBOT is most pronounced in the non-necrotic metabolic dysfunctional regions of the brain, even years after the acute insult (14).

The present report has several limitations. In this study, the treatment protocol included physical and occupational therapy twice a week during the HBOT course of treatment. The patient was in stable condition prior to treatment; in the absence of a control group, it is not clear whether the improvement in motor function is due to HBOT or due to the combined therapy even though there was no improvement with physical therapy before 2 years HBOT was initiated. The reliability of functional imaging analysis (task-based and rsFC) at the subject level is affected by many technical and personal factors. Although we used global brain parameters to describe changes and to reduce measurement error, motor deficits following a stroke are influenced not only by regional anatomical damage but also by the effectiveness of the rehabilitation process (51), and further investigations using a larger sample are needed to examine the potential of HBOT in the late chronic post-stroke stage.

In conclusion, our findings provide additional insights into how HBOT induces brain plasticity and functional improvement in chronic post-stroke patients. This study highlights the potential of a complementary analysis of rsFC and task-based imaging as tools for rehabilitation efficacy monitoring.

## Data availability statement

The original contributions presented in the study are included in the article/Supplementary material, further inquiries can be directed to the corresponding author.

## Ethics statement

The studies involving human participants were reviewed and approved by Shamir Medical Center Institutional Review Board (IRB). The patients/participants provided their written informed consent to participate in this study. Written informed consent was obtained from the individual(s) for the publication of any potentially identifiable images or data included in this article.

## Author contributions

MC and SE: conception and design. MC, MA, VE, and YP: investigation. MC: drafting the manuscript. MC, AH, YP, and SE: review and editing. All authors contributed to the article and approved the submitted version.
