# Supplementary material for: Functional MRI evaluation of hyperbaric oxygen therapy effect on hand motor recovery in a chronic post-stroke patient: a case report and physiological discussion
Source: Front Neurol. 2023 Sep 29;14:1233841. doi: 10.3389/fneur.2023.1233841 (PMC10570419; doi:10.3389/fneur.2023.1233841)
Supplement: Supplementary file 1 [file Data_Sheet_1.pdf]

Functional MRI evaluation of hyperbaric oxygen therapy effect on hand motor recovery in a chronic post-stroke patient: a case report and physiological discussion

Supplementary material

1 Supplementary figures 2

Figure 1. fMRI accusation system 2

Figure 2. Experimental design 3

Figure 3. Timeline 4

2 Supplementary tables 5

Table 1: Patient characteristics at enrollment (November 2021) 5

Table 2. Functional connectivity networks with regions of interest 6

Table 3. Fugl-Meyer upper extremity results 7

Table 4. Motor task fMRI - peak activation cluster analysis 8

1 Supplementary figures

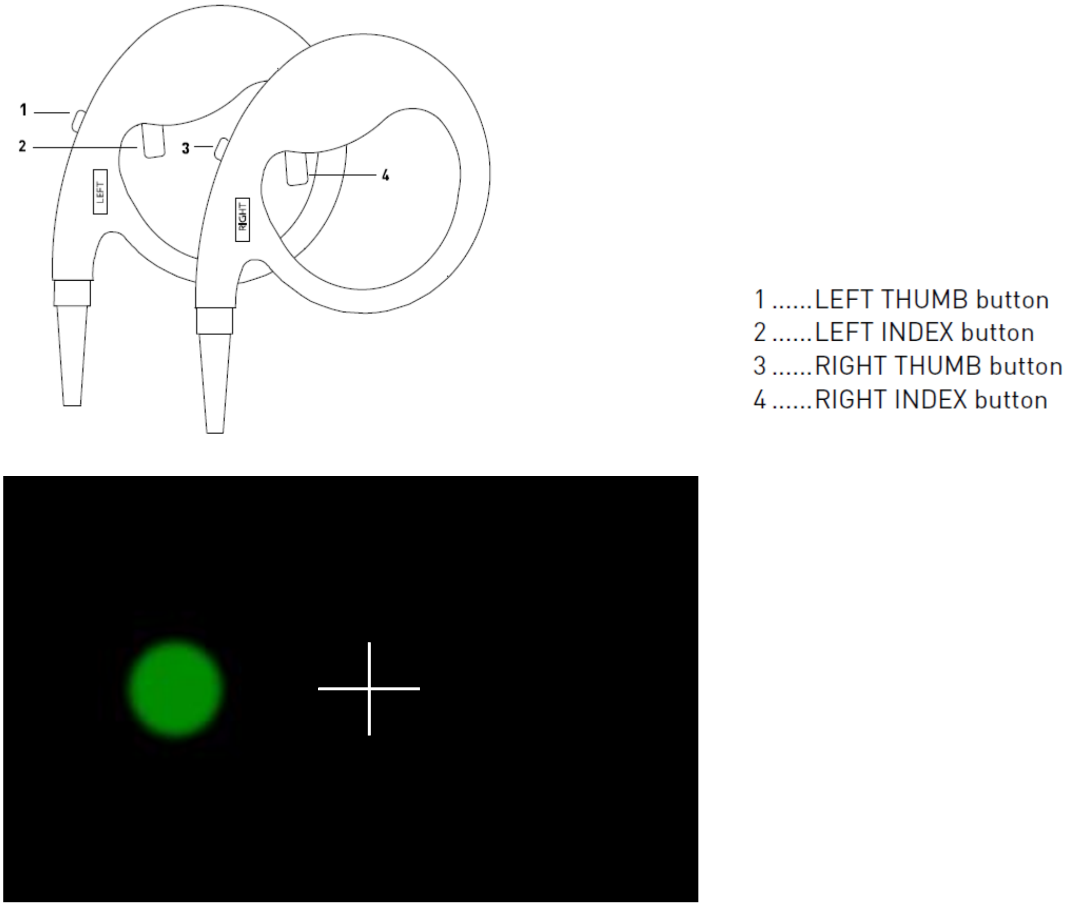

Figure 1. fMRI accusation system: (a) Hand-held grips, two response buttons per hand (ResponseGrip, NordicNeuroLab Inc., Norway). (b) Stimuli were displayed via a back-projection screen placed at the head of the scanner bore (Avotec Inc., Stuart, FL, USA; resolution: 800 × 600; refresh rate: 60 Hz), which is viewed by each participant via a mirror attached to the table near the subject's head.

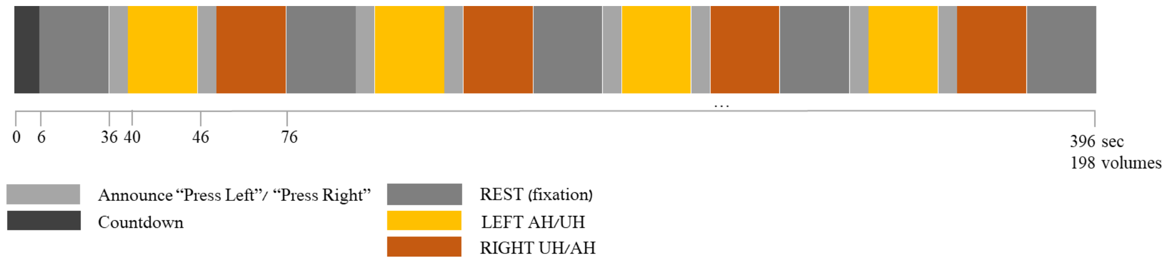

Figure 2. Experimental design. The test consists of ten 30 sec blocks: REST, LEFT, and RIGHT for the affected hand (AH) and unaffected hand (UH) responses to a flashing green dot presented either on the left or right side of the screen.

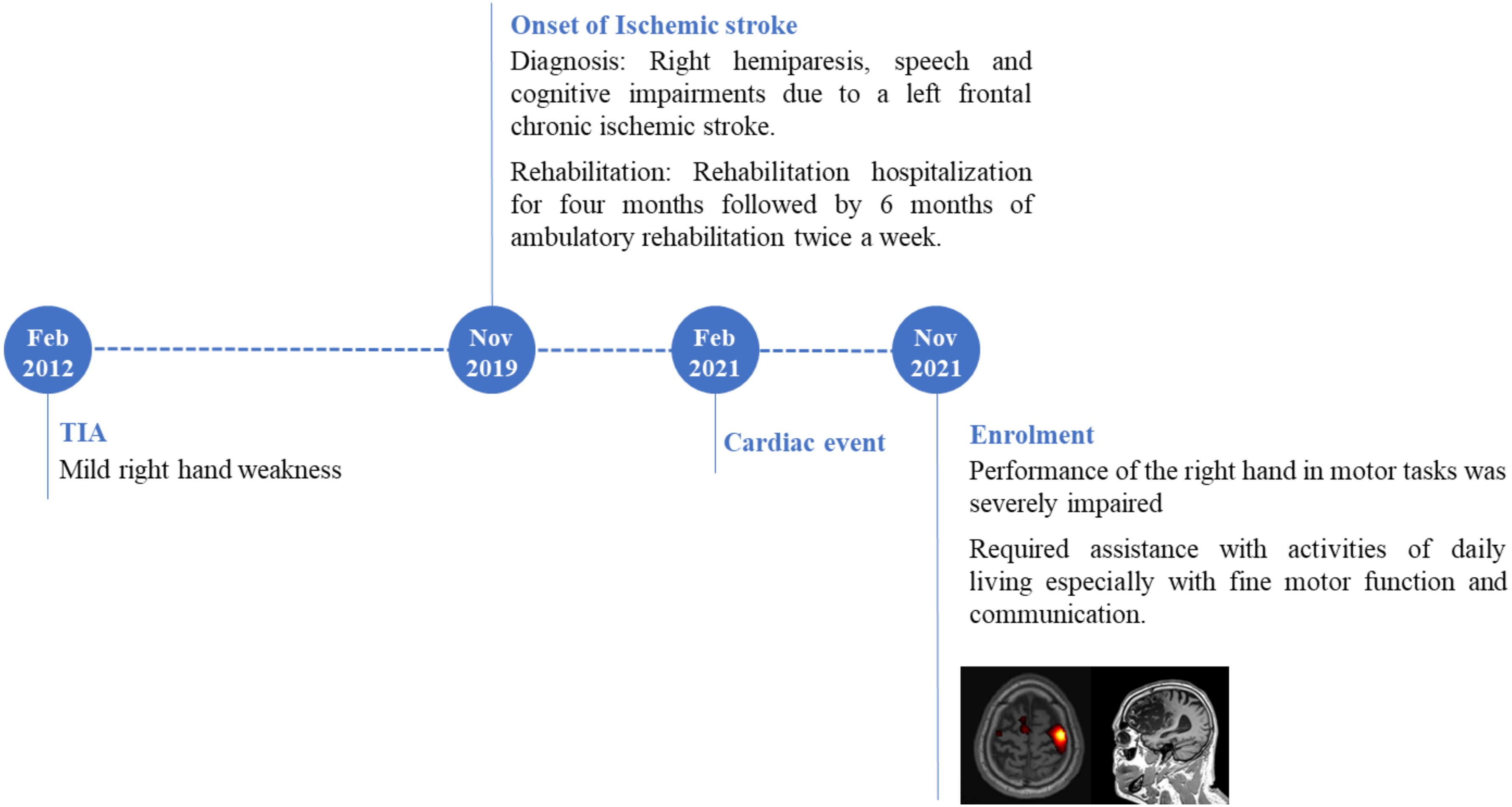

Figure 3. Timeline and relevant clinical information.

2 Supplementary tables

Table 1: Patient characteristics at enrollment (November 2021)

Age	61
Sex	Male
Marital status	Married
Number of children	3
Years of education	15
Occupation	Banker (for 38 years before the stroke)
Employment status	Unemployed
Family history of stroke	None
Handedness	Right
Co-existing medical conditions	Diabetes mellitus, hyperlipidemia, hypertension, and ischemic heart disease (February 2021)
Current medications	FERRIFOL, LEVITIRACETAM, ASPIRIN, ATOZET, PLAVIX METFORMIN
Smoking	No
Relevant genetic or psychosocial history	None
Onset of Stroke	November 2019

Table 2. Functional connectivity networks with regions of interest and center of mass coordinates

Resting-state networks	ROI	MNI coordinates*		
		X	Y	Z
Default Mode Network	Medial Prefrontal Cortex (MPFC)	1	55	-3
	Left Lateral Parietal (LP)	-39	-77	33
	Right Lateral Parietal (LP)	47	-67	29
	Posterior Cingulate Cortex (PCC)	1	-61	38
Sensorimotor Network	Right Lateral Area	-55	-12	29
	Left Lateral Area	56	-10	29
	Superior Area	0	-31	67
Salience Network	Left Anterior Insular Cortex (AInsula)	-44	13	1
	Right Anterior Insular Cortex (AInsula)	47	14	0
	Left Rostral Prefrontal Cortex (RPFC)	-32	45	27
	Right Rostral Prefrontal Cortex (RPFC)	32	46	27
	Left Supramarginal Gyrus (SMG)	-60	-39	31
	Right Supramarginal Gyrus (SMG)	62	-35	32
Dorsal Attention Network	Left Frontal Eye Fields (FEF)	-27	-9	64
	Right Frontal Eye Fields (FEF)	30	-6	64
	Left Intraparietal Sulcus (IPS)	-39	-43	52
	Right Intraparietal Sulcus (IPS)	39	-42	54
Fronto Parietal Network	Left Lateral Prefrontal Cortex (LPFC)	-43	33	28
	Left Posterior Parietal Cortex (PPC)	-46	-58	49
	Right Lateral Prefrontal Cortex (LPFC)	41	38	30
	Pight Posterior Parietal Cortex (PPC)	52	-52	45
Language Network	Left Inferior Frontal Gyrus (IFG)	-51	26	2
	Right Inferior Frontal Gyrus (IFG)	54	28	1
	Left posterior Superior Temporal Gyrus (pSTG)	-57	-47	15
	Right posterior Superior Temporal Gyrus (pSTG)	59	-42	13
Visual Network	Medial	2	-79	12
	Occipital	0	-93	-4
	Left lateral	-37	-79	10
	Right lateral	38	-72	13
Cerebellar Network	Anterior	0	-63	-30
	Posterior	0	-79	-32

* MNI coordinates correspond to network nodes provided by the CONN toolbox (v.18b) and obtained from the Human Connectome Project [1].

Table 3. Fugl-Meyer upper extremity results

Right hand (affected)	Scale	PRE	POST	1 Year Follow Up
A. Upper extremity	[0..36]	12	17	22
B. Wrist	[0..10]	0	3	5
C. Hand	[0..14]	5	10	13
D. Coordination/ Speed	[0..6]	0	1	1
**Total A-D**	[0..66]	**17**	**31**	41
H. Sensation	[0..12]	5	12	12
I. Passive joint motion	[0..24]	18	18	18
J. Joint pain	[0..24]	14	22	20

Table 4. Motor task fMRI - peak activation cluster analysis

Cluster size	Brain area	R/L	BA	T	X	Y	Z
**Left (UH) pre-intervention**							
2671	PMA	R	6	17.18	42	-16	64
	M1	R	4	11.84	40	-20	52
	S1	R	1	9.05	56	-18	44
152	PMA	R	6	7.48	-42	-10	52
99	SMA	L	6	6.78	-6	-2	56
53	SupGyrus	R	40	6.63	54	-14	16
57	PMA	R	6	5.73	54	2	34
20	CB	L		5.05	-28	-54	-54
**Left (UH) post-intervention**							
5359	PMA	R	6	18.83	40	-16	64
	S1	R	1	11.92	58	-20	46
	S1	R	1	11.74	40	-20	48
4251	CB	L		13.46	-24	-64	-18
702	Broca	R	44	9.70	58	16	-3
	Broca	R	44	9.33	58	12	20
1299	CB	R		9.51	52	-52	-32
	CB	R		8.88	26	-76	-20
	CB	R		8.55	38	-64	-22
365	AntPFC	R	10	9.12	30	54	22
	DLPFC	R	9	6.60	44	38	24
56	AngGyrus	L	39	7.93	-56	-54	12
52	AngGyrus	L	39	7.22	-56	-66	30
61	Broca	R	45	7.04	30	30	2
128	PMA	L	6	6.75	-42	-10	52
**Right (AH) pre-intervention**							
727	PMA	L	6	9.86	-30	-20	63
	S1	L	1	9.73	-46	-20	60
	M1	L	4	5.88	-44	-28	60
35	PPC	L	7	5.79	-20	-42	78
8	CB	R		5.09	54	-48	-34
**Right (AH) post-intervention**							
1582	PMA	L	6	15.66	-32	-20	70
	M1	L	4	15.37	-45	-18	62
	M1	L	4	12.99	-36	-18	54
791	CB	L		13.69	-24	-64	-18
	CB	L		8.99	-36	-54	-30
1134	Broca	R	44	11.09	56	16	0
2669	CB	R		10.85	2	-74	-36
	CB	R		10.78	26	-76	-20
885	SMA	L	6	10.52	-4	-6	54
	SMA	R	6	8.31	8	8	46

	SMA	R	6	8.22	10	4	60
800	PMA	R	6	10.51	48	0	48
	S1	R	1	8.17	64	-18	38
119	PMA	R	6	10.12	20	-8	58
214	AntPFC	R	10	9.90	32	50	20
230	SupGyrus	R	40	9.14	58	-14	16
147	CB	L		7.69	-34	-44	-54
	CB	L		6.35	-24	-40	-46
73	PPC	L	7	7.59	-20	-42	76
95	CB	R		7.48	30	-40	-40
136	SupGyrus	R	40	7.09	52	-34	26
162	AntPFC	R	10	7.06	40	58	0
	AntPFC	R	10	5.63	42	46	-10
50	AntPFC	L	10	5.92	-22	62	-8
	AntPFC	L	10	5.42	-16	66	-4

M1, primary motor cortex, SMA, supplementary motor area, PMA, premotor cortex, S1, primary somatosensory cortex, CB, cerebellum, AngGyrus, angular gyrus, DLPFC, dorsolateral prefrontal cortex, AntPFC, anterior prefrontal cortex, PPC, posterior parietal cortex, PFWE<0.05, L, left, R, right, FWE, familywise error, UH, unaffected hand, AH, affected hand, HC, healthy control, X, Y, Z MNI coordinates
